# Time-resolved metabolomics analysis of individual differences during the early stage of lipopolysaccharide-treated rats

**DOI:** 10.1038/srep34136

**Published:** 2016-10-03

**Authors:** Die Dai, Yiqiao Gao, Jiaqing Chen, Yin Huang, Zunjian Zhang, Fengguo Xu

**Affiliations:** 1 Key Laboratory of Drug Quality Control and Pharmacovigilance (China Pharmaceutical University), Ministry of Education, Nanjing 210009, China; 2 Jiangsu Key Laboratory of Drug Screening, China Pharmaceutical University, Nanjing 210009, China; 3 State Key Laboratory of Natural Medicine, China Pharmaceutical University, Nanjing 210009, China

## Abstract

Lipopolysaccharide (LPS) can lead to uncontrollable cytokine production and eventually cause fatal sepsis syndrome. Individual toxicity difference of LPS has been widely reported. In our study we observed that two thirds of the rats (24/36) died at a given dose of LPS, while the rest (12/36) survived. Tracking the dynamic metabolic change in survival and non-survival rats in the early stage may reveal new system information to understand the inter-individual variation in response to LPS. As the time-resolved datasets are very complex and no single method can elucidate the problem clearly and comprehensively, the static and dynamic metabolomics methods were employed in combination as cross-validation. Intriguingly, some common results have been observed. Lipids were the main different metabolites between survival and non-survival rats in pre-dose serum and in the early stage of infection with LPS. The LPS treatment led to S-adenosly-methionine and total cysteine individual difference in early stage, and subsequent significant perturbations in energy metabolism and oxidative stress. Furthermore, cytokine profiles were analyzed to identify potential biological associations between cytokines and specific metabolites. Our collective findings may provide some heuristic guidance for elucidating the underlying mechanism of individual difference in LPS-mediated disease.

Lipopolysaccharide (LPS), an integral component of the Gram-negative bacterial outer membrane, can lead to uncontrollable cytokine production, which can result in cardiovascular collapse and hemodynamic instability, and can eventually cause fatal sepsis syndrome^1^,^2^. Early recognition of septic shock and rapid treatment in the first golden hours after the diagnosis is important for a positive outcome^3^. New diagnostic tools are needed to accelerate diagnosis and improve prognosis. Increasing evidences^4^,^5^,^6^ suggest that the genetic variability in toll-like receptor 4 (TLR4)-mediated LPS responses play a crucial role in determining the outcome of infectious disease. Pharmacogenetics provides a powerful tool to understand the genetic variants, but its impact on medicine is challenged by the genotype-environmental interactions^7^. Metabolomics^8^, a crucial element in bridging the difference between the genotype and phenotype, is increasingly applied to identify and validate targets to improve individualized health^9^.

Conventionally, a biomarker in metabolomics is sought by comparing differences in the metabolic profiles between two states (e.g. healthy *vs* pathological)^10^. Clear evidence has been generated that biomarker patterns of living systems on a single time point provide important information but do not capture the dynamics of a system^11^,^12^. Metabolism is a dynamic process, controlled through basic kinetic regulatory mechanisms, where the overall system aims to maintain a state of homeostasis. So it is very important to monitor the dynamic metabolic changes in response to disease development. However, datasets resulting from time-series studies are very complex and contain multiple types of variation^13^: the variation originating from differences between the animals that are constant in time, the time-dynamic variation of each individual animal, or combinations thereof. It is necessary to incorporate this structure into the data analysis for a deep understanding the biological information in these datasets. Meanwhile, in time-course studies, the observation at different time points and from different replications can be correlated, which is quite different from the static studies and may increase the risk of false positives and false negatives.

A number of methods, sometimes borrowed from other disciplines, have been proposed for analysis of time-resolved metabolomics data^12^. Analysis of variance (ANOVA) Simultaneous Component Analysis (ASCA), a direct generalization of ANOVA for univariate data to the multivariate case, can deal with such multilevel datasets^14^. The variation splitting property of ANOVA allows a separate analysis and interpretation of the variation sources induced by the different factors in the experimental design^15^. By taking into account any correlations among observations at different times and the replication, multivariate Bayesian time-series analysis (MEBA) can identify the metabolites whose expression varied the most across each developmental time point^16^. Additionally, batch process (BP), has proven to be a powerful metabolomic tool in defining time-dependent metabolic consequences of toxicity^17^ and is an efficient means of visualizing inter-animal variations in response^18^, with each rat treated as a separate batch.

We previously observed the individual toxicity difference of LPS. Two thirds (24/36) of the rats (non-survival group, NS) died at a given dose of LPS, while the rest (12/36) survived (survival group, S). A pharmacometabonomic analysis of the pre-dose serum was carried out. The results indicated that the inter-subject difference before LPS treatment was mainly associated with lipid metabolism^19^. To further clarify the individual toxicity difference more systematically between the survival and non-survival rats in the early stage after LPS administration, the time-resolved metabolomics analysis were conducted from multiple perspectives. First, the typical static metabolomics analysis on a single time point was performed. As the survival and non-survival rats have the same reference, the SUS-plot (shared and unique structure) was used for the evaluation of the potential biomarkers^20^. Then, three multiple statistical methods (ASCA/MEBA/BP) with different principle were used to monitor the dynamic metabolic changes in response to disease development. Furthermore, cytokine profiles were analyzed to identify potential biological associations between cytokines and specific metabolites. Our collective results, to the best of our knowledge, are the first to comprehensively demonstrate the individual toxicity difference in the early stage after LPS administration and provided insight into the mechanism of LPS-mediated disease.

## Results

### Animal models

**Figure 1:**
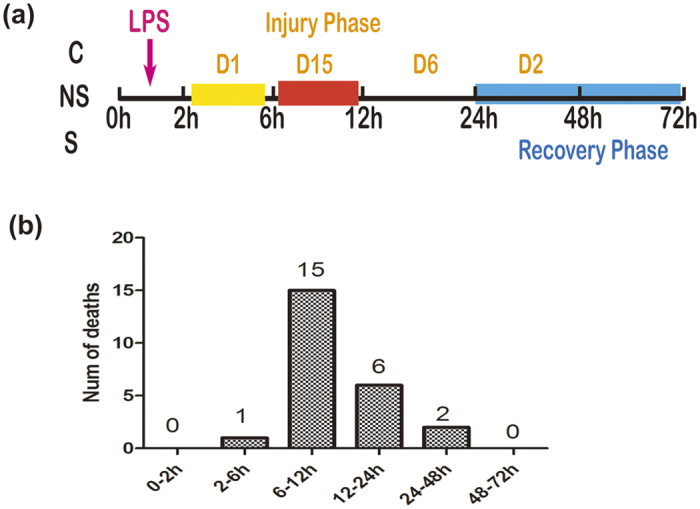
(**a**) Scheme of the animal experiment protocol in the present study. D1: a rat died from 2 h to 6 h; D15: 15 rats died from 6 h to 12 h; D6: 6 rats died within 12 h to 24 h; D2: 2 rats died from 24 h to 48 h. (**b**) The LPS group (n = 36) animals were injected with LPS (10 mg/kg) and mortality was monitored for 72 h.

All LPS-challenged rats produced signs of endotoxemia, such as lethargy, piloerection, and diarrhea within 30 min after LPS injection, and a gradual physiological recovery was observed in survival rats after 12 h. However, these pathological features were absent from control rats. In the LPS group, 24 rats died during the first 72 h after LPS treatment, while 12 rats survived. Among those deaths, 15 rats died from 6 h to 12 h and 6 rats died between 12 h and 24 h, we infer that rats suffered a serious injury during this phase (Fig. 1). The mortality was very low after 24 h and rats may begin to recover.

### Data acquisition quality

All serum samples from LPS-treated and the control rats were randomized in order to avoid batch effects. Quality control (QC) samples were prepared by pooling equal aliquots of serum from each sample. The first nth QCs (n = 5 for GC–MS and n = 10 for LC–MS) were tested before the analysis to stabilize the analytical system^21^ and the acquired data were removed prior to data processing.

In order to monitor the robustness of sample preparation and the stability of instrument analysis, QC samples were intermittently injected through the analytical experiment.

Total area normalization was chosen as previous research has shown this as “fit for purpose” for multivariate statistical data analysis. In case of GC-MS (gas chromatography-mass spectrometry) and LC-MS (ESI-) (liquid chromatography-mass spectrometry and mass spectrometry with negative electrospray ionization) data, tight clustering of the QCs in the PCA (principle component analysis) scores were observed, indicating good reproducibility of the data (Supplementary Fig. S1b,c). However, in case of the LC-MS (ESI+) data, analytical variation associated with interbatch measurements across multiple days were observed (Supplementary Fig. S1a), which we addressed with a feature-based signal correction (FBSC) algorithm using the QC intensity values in the local neighborhood of each sample to formulate a correction factor^22^. The new normalization method showed a remarkable improvement in the clustering of the QCs (Supplementary Fig. S1d), suggesting an acceptable run providing data suitable for further statistical analysis. An additional insight into trends and drifts within the whole analytical run according to sequence order is shown in Supplementary Fig. S1e, which depicts the first component t [1] versus the samples. The results showed that the detection system was stable throughout the experiment, with no outliers exceeding ±2 SD (standard deviation) in the QC samples.

### Serum metabolomic trajectory analysis in rats

**Figure 2:**
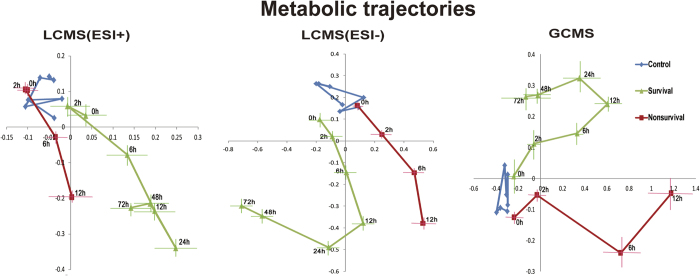
The time-dependent trajectory of the metabolic profiles of the control and the LPS groups. Each dot represents the average metabolic status at a certain time; bar lines indicate the SEs of PC1 and PC2 in the PCA model.

After aligning peaks, 1854, 225, and 156 spectral features were obtained from each sample analyzed by GC/MS, LC/MS (ESI+) and LC/MS (ESI−), respectively. To investigate the global metabolomic alterations in rats, all observations acquired in both ion modes were analyzed using PCA and the mean PC (principal component) scores trajectories were calculated for the first two PCs. The time-course trajectories (Fig. 2) showed characteristically different paths for each group. The data points of the control group did not separate from each other during the time course. While the model groups (both the S and NS groups) exhibits a response of great magnitude. The degree of movement away from pre-dose i.e., the magnitude of metabolic effect, was much greater in 12 h than in the other time points after LPS treatment. The survival trajectory appears to recover at 24 h while the non-survival trajectory was stop at 12 h. A clear separation between the S and NS groups was observed in the PCA score plot before LPS treatment (0 h) and in the progression of the induced toxic lesions. These constructed metabolic trajectories were consistent with the results of the mortality rates, showing severe disturbances in metabolic profile corresponding to the maximal time of damage (6 h~24 h). However, the difference in the early stage (0~6 h) of the damage between the S and NS may give more information for the explanation of inter-individual variations. So we focus on this stage. As the three-way dataset (multi-subjects, multi-time points and multi-variables) generated in the time-resolved metabolomics (Supplementary Fig. S2) is very complex and no single method can elucidate the problem clearly and comprehensively, a combined method including the static and dynamic metabolomics from multiple perspectives has been conducted here.

### Typical static metabolomics analysis of the inter-individual differences at the early stage (0~6 h) after LPS treatment

**Figure 3:**
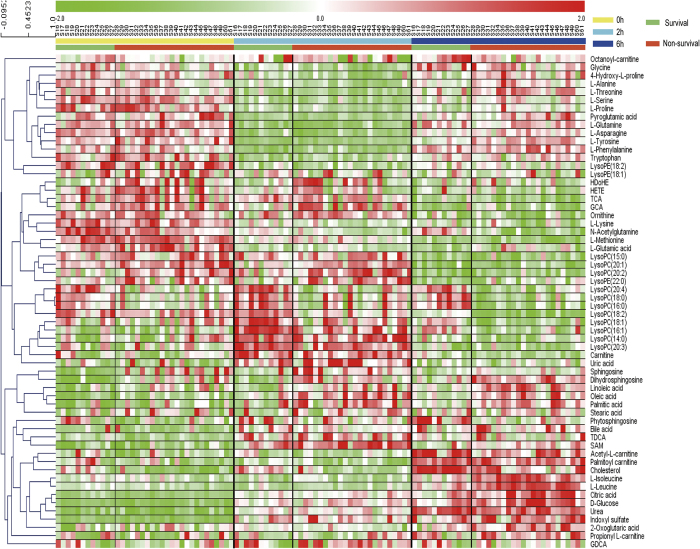
A heatmap representing the metabolic changes in survival and non-survival rats within 0 h to 6 h. Green square indicates a reduction of up to two folds, white square indicates no significant fold change, and red square indicates an increase of up to two folds.

Among the features, 60 metabolites were finally putatively validated and listed in Supplementary Tables S1 and S2. A heatmap (Fig. 3) was constructed to present the metabolic alterations of these identified metabolites after LPS intervention. The data was reordered in the rows to group the similar data together by hierarchical clustering, as shown by dendrograms for the rows. The metric for clustering is Pearson correlation. It could be observed that most of amino acids were reduced in both survival and non-survival rats at 2 h after LPS treatment, and the up-regulations were obvious at 6 h. Additionally, subsets of metabolites, mainly involved in tricarboxylic acid (TCA) cycle, urea cycle, were elevated at 6 h. Furthermore, it was clear that lipids, the main different metabolites between survival and non-survival rats in pre-dose serum, were still different in the two groups at 2 h and 6 h after LPS administration.

**Figure 4:**
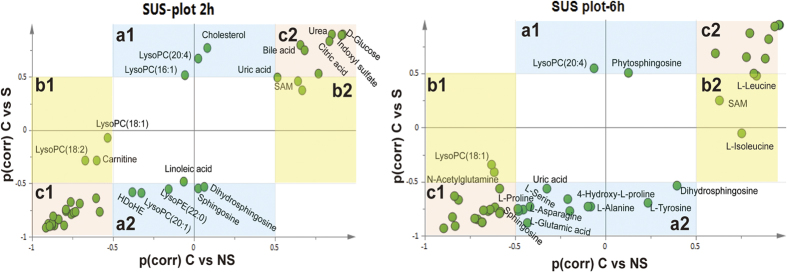
SUS plot of survival and non-survival rats at 2 h and 6 h, respectively. All metabolites not significant (|p(corr)| <0.5) in either model were removed in this plot to enhance visualization.

To compare survival and non-survival rats against control rats as common reference, SUS plots were used. As shown in Fig. 4, the variables located in region “a1” or region “a2” were only up- or down- regulated in survival rats, reflecting the metabolic character of the survival group. Conversely, the variables located in region “b1” or region “b2” were specific to the non-survival rats. The variables located on the diagonals (region “c1” or region “c2”) were important for both models. Details of these compounds were given in Supplementary Table S3. As a result, metabolites including S-adenosly-methionine (SAM), carnitine and lysophosphatidylcholines (lysoPC(18:1) and lysoPC(18:2)) at 2 h and leucine and isoleucine at 6 h were identified to be specific to non-survival rats. The unique identified metabolites for survival rats at 2 h were mainly involved in lipid metabolism. Several metabolites involved in energy metabolism, urea cycle, oxidative stress and bile acid metabolism were shared by both survival and non-survival rats.

### Inter-individual differences at the early stage (0~6 h) after LPS treatment analyzed by the dynamic metabolomics—ASCA and MEBA

**Figure 5:**
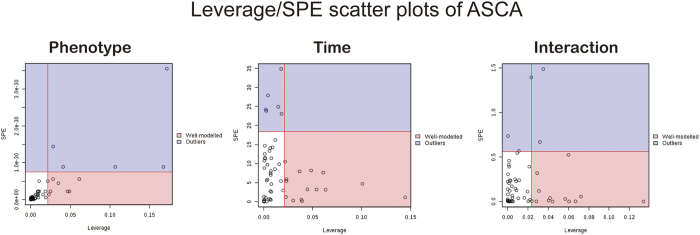
Leverage/SPE scatter plots of the ASCA variables submodels for phenotype, time and their interactions. Metabolites in red have high loadings that follow the expression patterns of the submodels. Metabolites in blue have expression patterns that are different from the major patterns.

For the multilevel datasets which contains multiple types of variation, ASCA was applied to split the original dataset into different subsets describing the variation between animals, the variation in time and their interaction. The dataset was first normalized to constant sum (Supplementary Fig. S3a) and transformed using the generalized log transformation and Pareto scaling, and then introduced into the MetaboAnalyst web portal^23^. As part of the first stage to determine the number of factors to extract, a scree plot (Supplementary Fig. S3b) was used which graphically displays the relationship between eigenvalues and factors. The major patterns associated with factor A (time variation), factor B (phenotypes, survival or non-survival) and their interaction were shown in the score scatter plots (Supplementary Fig. S3c) based on PC1 of the corresponding sub models. In a follow-up process, a significance test (Supplementary Fig. S3d) was performed by a permutation approach^24^ to validate the model, as demonstrated by significance levels of *p* < 0.05 for the phenotype, time level, and their interaction. The significant variables associated with a specific factor were identified based on the leverage/squared prediction error (SPE) plots (Fig. 5). Supplementary Table S4 shows features well-modelled by phenotype, time and their interaction. A total of 15 metabolites were greatly altered over time, such as indoxyl sulfate, D-glucose, bile acid and urea. It was interesting that SAM, identified to be specific to non-survival rats at 2 h in SUS-plot, was selected again as a significant variable associated with phenotype (survival or non-survival).

The time-course profiles of representative metabolites with high rankings by MEBA were shown in Supplementary Fig. S4. Among these metabolites, sphingosine, dihydrosphingosine, oleic acid, lysoPC(18:2) and lysoPC(20:4) are involved in lipid metabolism, and alanine, glucose and citric acid are involved in energy metabolism. In addition, time profiles of leucine, isoleucine and SAM were selected again to be significant different between survival and non-survival rats.

### Batch process for the inter-individual differences at the early stage (0~6 h) after LPS treatment and the sub-models of the non-survival rats

Batch process usually operates on two levels, with each rat treated as a separate batch. At the lower level, a PLS (partial least squares) time model was constructed initially using all survival rats in order to derive optimal metabolic information for survival samples. This resulted in a strongly significant 3 component PLS model with R^2^X = 0.554, R^2^Y = 0.949 and Q^2^Y = 0.897. The average trajectory for the evolution of survival batches from pre-dose (0 h) to 6 h post-dose was represented by a solid black line with upper and lower control limits (±1.5 SD in this study) being represented by dashed black lines (Supplementary Fig. S5a). The non-survival rats were then predicted individually into the survival model to establish metabolic deviations from the survival. PLS scores vectors (t [1]–t [3]) were plotted with time to describe the different phases of the dominant metabolic perturbations with time. The reason for the deviations from batch normality can be found in the PLS contribution plots (Supplementary Fig. S5b). Although the PLS vector directions described by t [1], t [2] and t [3] were different, it was interesting that the metabolites relating to these deviations at the same sampling time were almost same in every component. The deviation observed at 0 h was predominantly attributed to lipids as described in our previous work. In addition to lipids, elevation in serum concentration of SAM was chiefly responsible for the deviation of non-survival profiles from the survival model at 2 h. Other significant perturbations at 6 h included the change of urea, glucose, citric acid, indoxyl sulfate and branched amino acid.

The upper-level PCA model consisted of two components describing 60.2% of the variation in X, based on the lower-level PLS scores. From the PC scores plot at the upper batch level, all 12 survival batches spanned the space with no outliers (Supplementary Fig. S6a), indicating that all the samples were well fitted to the survival model. Using this model to predict the 24 non-survival rats, intriguingly, rats died at 12 h and 24 h (marked with red circle) were found to fit the model and some of the rats died within 6 h lay outside the Hotelling T2 ellipse (Supplementary Fig. S6b). This provided a scale of the magnitude of response for each animal and indicated that the non-survival rats could still be subdivided into “fast” or “slow” responders to LPS. Further, PCA and OPLS-DA (orthogonal partial least squares-discriminant analysis) models were used to explore the difference between the “fast” (NS1group) and the “slow” (NS2 group) responders of the non-survival rats (Supplementary Fig. S6c–h). A clear separation between these two groups was observed and the deviation was predominantly influenced by the different serum concentration of lipids (Supplementary Table S5). The PCA and the OPLS-DA modeling were summarized in Table 1.

### Quantification of total thiols in serum

Intriguingly, apart from lipids, SAM was identified again and again as an early differential metabolite by both the static (SUS-plot) and dynamic (ASCA, MEBA and BP) methods. Our initial plan is to accurately quantify SAM for a further validation. Unfortunately, SAM is unstable in biological samples^25^,^26^ and was not detectable in serum after long-time storage in our study. As SAM can be converted to cysteine and glutathione via trans-sulfuration pathway^27^, so an indirect validation of a quantitative retrospective assessment of total thiols (total cysteine (Cys), glutathione (GSH), N-acetylcysteine (NAC), cysteamine (CA), homocysteine (Hcy), γ-glutamyl-cysteine (γ-Glu-Cys) and cysteinyl-glycine (Cys-Gly)) was then performed. Representative MRM (multiple reaction monitoring) chromatogram of each analyte in a serum sample of control rats was shown in Supplementary Fig. S7. Linearity of each analyte in serum using the proposed method was listed in Supplementary Table S6. As shown in Supplementary Fig. S8, total cysteine was significantly increased in the non-survival group at the early stage (2 h), while other compounds showed no significant differences between the two groups.

### Serum concentrations of cytokine/chemokine and its associations with metabolites

**Figure 6:**
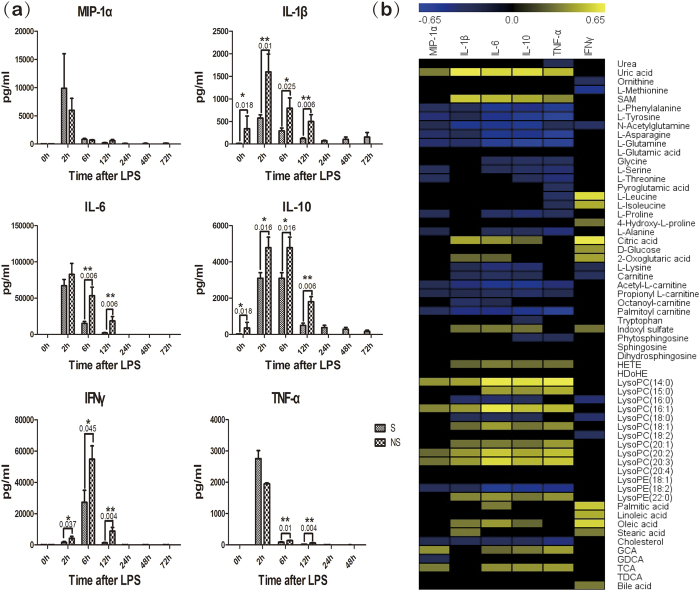
(**a**) Time course of serum concentrations of a panel of cytokines. MIP, macrophage in flammatory protein. The nonparametric Mann-Whitney test was employed to assess the statistical significance between S and NS (n = 8). (*)*p* < 0.05, (**)*p* < 0.01. (**b**) A correlation heatmap of satistical associations of metabolites to cytokines. Blue squares indicate significant negative correlations, black squares indicate nonsignificant correlations (p > 0.05), and yellow squares indicate significant positive correlations.

The data for 6 cytokines (macrophage inflammatory protein (MIP)-1α), interleukin (IL)-1β, IL-6, IL-10, tumor necrosis factor (TNF)-α and interferon (IFN)-γ) in the serum of survival and non-survival rats was summarized in Fig. 6a. Serum concentrations of these cytokine in survival rats increased transiently after LPS administration, but there were specific temporal patterns of the responses for different cytokines. Concentrations of MIP-1α, IL-1β, IL-6 and TNF-α all reached peak concentrations at 2 h after LPS administration and then declined. Concentrations of IL-10 reached peak values at 2 h and 6 h. And concentrations of IFNγ was slightly elevated by 2 h, reached a peak value at 6 h, and decreased to baseline by 24 h. The change of the cytokines concentrations in non-survival rats was the same as the survival ones, but was different in degree.

Pearson correlation analysis (Fig. 6b) was used to identify potential links between metabolites and cytokines. MIP-1α, IL-1β, IL-6, IL-10, TNF-α and IFNγ were determined to correlate negatively with L-phenylalanine, L-tyrosine, nitrogen metabolism (N-acetylglutamine, L-asparagine, L-glutamine) and carnitine derivatives. These cytokines were also statistically linked positively to uric acid, SAM, citric acid, 2-oxoglutaric acid and lysophosphatidylcholines levels.

## Disscusion

**Figure 7:**
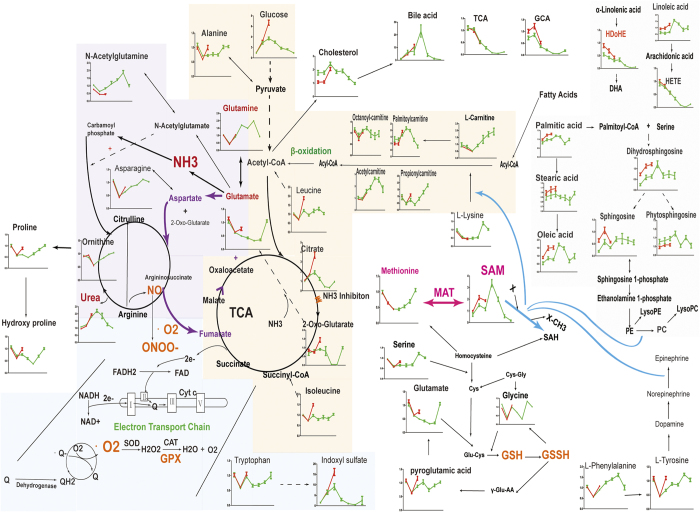
Schematic overview of the time course analysis of the survival and non-survival rats.

To elucidate the possible underlying mechanism of the individual toxicity difference between the survival and non-survival rats after LPS administration, a hypothetical metabolic network using the metabolites annotated (Supplementary Tables S1 and S2) was reconstructed referring to the Kyoto Encyclopedia of Genes and Genomes, MetaCyc^28^, the Human Metabolome Database^29^ and other literatures. As shown in Fig. 7, the time course analysis of the survival and non-survival rats indicates that the inter-subject difference in the early stage is associated with multiple metabolic pathways.

Lipids, the main different metabolites between survival and non-survival rats in pre-dose serum, were still different in the two groups at 2 h and 6 h after LPS administration. Lipids were also the main different metabolites between the “fast” and the “slow” responders of the non-survival rats. Furthermore, they were statistically linked positively to cytokines. Lipids play a significant role in individualized response to LPS and a detailed discussion about the links between lipids metabolism and LPS susceptibility has been reported in our previous work^19^.

Intriguingly, SAM was identified as an early differential metabolite by both the static and dynamic methods. As an important molecule in normal cell function and survival, SAM is synthesized from adenosine triphosphate (ATP) and methionine (Met) in two consecutive reactions by methionine adenosyltransferase (MAT)^30^. SAM has three possible fates^27^: (a) transfer of its methyl group to a variety of methyl acceptors like DNA (deoxyribonucleic acid), phospholipids and proteins, affecting a wide spectrum of processes ranging from gene expression to membrane fluidity, (b) conversion to cysteine via trans-sulfuration pathway, as a precursor of GSH, the major cellular anti-oxidant, and (c) decarboxylation followed by aminopropylation leading to polyamine synthesis. Emerging data^31^,^32^ indicated that hepatic SAM deficiency is associated with increased susceptibility to LPS-induced hepatotoxicity, and administration of exogenous SAM attenuates these effects. Unexpectedly, we found SAM level increased and was higher in NS rats at 2 h after LPS administration, suggesting an increase in SAM biosynthesis and/or block in transmethylation. Kwangsuk Ko *et al.*^33^ found the same results. They suggested that hepatic SAM level increased 67% following LPS treatment while S-adenosylhomocysteine level fell by 26% and GSH is markedly depleted. Additionally, many immunologic functions are highly dependent on methylation events^34^. TLR4 is the pattern-recognition receptors for LPS^35^ and TLR signaling was found to be transmethylation-dependent^36^. Here, our investigation also reveals strong associations between SAM alterations and cytokines.

Glucose and citrate, involved in TCA cycle and energy metabolism, were increased at 2 h and reached its highest value at 6 h. It is worthy to remark that the levels of glucose, citrate and 2-oxoglutaric acid in non-survival rats were significantly higher than in the survivals at 6 h (Supplementary Table S7). Branched-chain amino acids (isoleucine and leucine), have some links to TCA cycle, were also increased significantly at 6 h in non-survival rats. In addition, citric acid and 2-oxoglutaric acid were statistically linked positively to cytokines. And the uncontrollable cytokine production caused by LPS can lead to fatal sepsis syndrome. The significant perturbations in TCA cycle at 6 h may be the direct cause of death.

Mitochondria generate most of the energy in animal cells because they link the energy-releasing activities of electron transport and proton pumping with the energy conserving process of oxidative phosphorylation^37^. NADH (reduced nicotinamide adenine dinucleotide) or FADH (flavine adenine dinucleotide, reduced), generated by associated TCA cycle dehydrogenases, can be oxidized and feeds electrons to the electron transport chain^38^. Oxidative stress^39^ is an unavoidable by-product of electron transport chain due to electron transfer to O_2_. As a significant perturbation in TCA cycle in non-survival rats at 6 h was observed, we infer that higher oxidative stress may have occurred at this time. To protect against the damage caused by oxidative stress, cells possess a sophisticated enzymatic and non-enzymatic antioxidant defence system^40^. GSH, a non-protein sulfhydryl containing compound, is one of the major intracellular regulator of redox homeostasis^41^. It is reported that LPS can inactivate hepatic MAT activity and glutathione is an important variable that determines susceptibility to LPS-induced liver injury^33^. SAM can be converted to cysteine and GSH via trans-sulfuration pathway. Our findings indicated that sulfur metabolism was genuinely different between survival and non-survival rats in the early stage of LPS administration. SAM and total cysteine were significantly increased in the non-survival group, while total GSH showed no significant differences between the two groups.

In conclusion, we have demonstrated a time-resolved metabolomics analysis of individual differences during the early stage of LPS-treated rats. A very rigorous statistical analysis (including the static and dynamic metabolomics), followed by extensive pathway mapping has been performed here. Metabolic profiling results were also supported by the analysis of a cytokine panel. The mechanism of the individual toxicity difference of LPS in rats is very complex, involved in lipid metabolism, sulfur metabolism and TCA cycle. These metabolisms are connected with each other. TCA cycle has some links with oxidative stress and growing evidence^42^ indicates that oxidative stress and lipid metabolism are also connected. Sulfur metabolism has the anti-oxidative capacity. As metabolism is a dynamic process, we infer that redox homeostasis may be disturbed after LPS challenge and death was eventually caused. However, these novel findings in our paper are within the discovery stage of metabolomics and thus generate hypotheses that should be investigated in a more targeted manner in the future. Moreover, as a limitation of this study, we investigated a small group of rats. Larger studies might allow for the validation of these hypotheses.

## Materials and Methods

### Chemicals and reagents

LPS (*E. coli* O55:B5) and all other chemicals were obtained from Sigma-Aldrich (St. Louis, MO, USA). Methanol, acetonitrile (HPLC grade) were purchased from Merck (Germany). Distilled water was filtered through a Milli-Q system from EMD Millipore Corporation (Billerica, MA, USA).

### Animal experiments and sample collection

All animal experiments were performed in accordance with the institutional guidelines for the care and use of laboratory animals. And all experimental protocols were approved by the Animal Ethics Committee of China Pharmaceutical University. A total of 50 male Sprague-Dawley rats (220 to 260 g body weight) were purchased from Vital River Laboratory Animal Technology Co., Ltd., Beijing, China. All animals were maintained under specific-pathogen-free conditions and were given a standard rat diet and tap water ad libitum for 1 week before experiments.

Animals were randomly assigned to a control or LPS group. The LPS group (n = 36) animals were injected intravenously with 10 mg/kg LPS. The chosen dose of LPS was based on our previous study^19^ and preliminary experiments. The control group (n = 14) animals were given an equivalent volume of sterile saline. Blood samples were drawn before (0 h) and 2 h, 6 h, 12 h, 24 h, 48 h and 72 h after administration of LPS. The time points were chosen according to the literature^43^ and our preliminary experiments, including both the acute and recovery phases of the response. On collection, blood samples were centrifuged at 5000 rpm for 10 min, and were subsequently stored at −80 °C before metabolomics analysis. Survivals were recorded for 72 h.

### Sample preparation for metabolomics analysis

Serum was thawed before preparation at room temperature. 240 *μ*L acetonitrile was added to 30 *μ*L serum for protein precipitation and then the mixture was vortexed thoroughly for 5 min. After two centrifugations (16,000 rpm, 10 min, 4 °C), the supernatant was divided into two aliquots. One was transferred into an autosampler vial and employed directly for UFLC-IT-TOF/MS (ultrafast liquid chromatography coupled with ion trap-time of flight mass spectrometry) analysis. The other (80 *μ*L) was transferred to a glass sampling vial and oximated with 25 *μ*L methoxyamine hydrochloride (10 mg/mL) in pyridine at 1200 rpm for 90 min at 37 °C. The mixture was then vacuum dried at 50 °C for 2 h (Labconco CentriVap®, Kansas, MO, USA). Later, 120 *μ*L of MSTFA (N-methyl-N-(trimethylsilyl) trifluoroacetamide) was added and kept for 2 h at 37 °C at 1200 rpm for trimethylsilylation, the supernatant was then separated for GC–MS analysis.

### UFLC–IT–TOF/MS spectral acquisition of serum samples

UFLC-IT/TOF-MS analysis was performed on a Shimadzu Prominence series UFLC system coupled to ion trap/time-of-flight hybrid mass spectrometry (Shimadzu, Kyoto, Japan) via electrospray ionization (ESI) source. Chromatographic separations were achieved using a Phenomenex Kinetex C18 column (100 × 2.1 mm, 2.6 *μ*m; Phenomenex, Torrance, CA, USA) equipped with a guard column, SecurityGuard ULTRA cartridge UHPLC C18. The column temperature was held at 40 °C. The mobile phase was a mixture of (A) H_2_O with 0.1% formic acid and (B) acetonitrile, with a programmed gradient as follows: linear gradient from 5% B to 95% B, 0–20 min; maintained with 95% B in 3 min, and returned to 5% B for column equilibration for 7 min. The injection volume was 5 *μ*L, and the flow rate was maintained at 0.4 mL/min.

Mass spectrometry operation parameters were set as follows: the mass range was scanned from 100 to 1000 m/z using an accumulation time of 20 ms per spectrum. Both positive and negative ion modes were used, with interface voltage at 4.5 kV and −3.5 kV, respectively. The curved desorption line (CDL) and heat block temperature were both 200 °C and the detector voltage of the TOF analyzer was 1.65 kV. Nitrogen was used as the nebulizer and drying gas, set at a constant flow rate of 1.5 L/min and 10 L/min, respectively. In the tandem mass spectrometry (MS/MS) experiments, argon was employed as the collision gas and collision energy was altered between 10 and 60 eV. Instrument calibration was performed with an external standard of sodium trifluoroacetate (STFA) solution. The data were recorded and processed by the LC/MS solution V3.41 software, including a chemical formula predict program (Formula Predictor). The identification of biomarkers followed a comprehensive strategy involving the determination of the accurate m/z, retention time, and typical MS/MS fragment and pattern. Clustering analysis (with Pearson correlation) was carried out to identify clusters of ions originating from the same metabolite (i.e., molecule ions, in-source molecular fragments, (de)protonated molecule ions, adducts ions and ^13^C isotopes). The chemical formulae were predicted by comparison of theoretical and observed m/z value results and isotopic patterns using Formula Predictor. Discriminative markers were then compared with the mass-to-charge ratio (m/z), formulae and the MS/MS fragmentation of metabolites proposed by literatures and freely available databases, namely the Human Metabolome (HMDB; http://www.hmdb.ca), the METLIN Metabolite (http://metlin.scripps.edu), the Mass Bank (http://www.massbank.jp) and the LIPID MAPS Structure Databases (http://www.lipidmaps.org).

### GC/MS Spectral acquisition of serum samples

All GC-MS analyses were carried out on a Shimadzu GCMS QP-2010 model gas chromatograph-mass spectrometer equipped with a fused silica capillary column (Rtx-5MS; 30 m × 0.25 mm (inner diameter), film thickness: 0.25 *μ*m; Restex). Helium was employed as the carrier gas at a constant flow rate of 1 mL/min and the injector split ratio was set to 1:50. The oven temperature was programmed at 70 °C for 2 min, followed by an increase to 320 °C at 10 °C/min and held at 320 °C for 2 min. The total run time was 29 min. The temperatures of the injector, transfer line and ion source were maintained at 250, 250, and 200 °C, respectively. The mass spectrometer was operated in electron impact mode (70 eV). Data acquisition was performed in full scan mode from m/z 45 to 600 with a scan time of 0.2 s. Identification of the compounds was done by comparison of mass spectra with those available in National Institute of Standards and Technology (NIST) library (version 2.0, developed by NIST for Shimadzu Co., Ltd) using a similarity index (SI) as a percentage for discrimination from nearest neighbors. Then those metabolites were further confirmed by comparing with the standards that available in our lab.

### Data processing

Raw data obtained from LC/MS and GC/MS analysis was input to Profiling Solution version 1.1 (Shimadzu, Kyoto, Japan) for peak deconvolution and alignment. Solvent blanks were run between samples and each mass is checked against the blank run to exclude possible sources of contaminations. Eighty percent filtering was applied to the raw data to ensure selection of relevant metabolites. Masses whose abundance was not reproducible for all biological replicates, as indicated by a Relative Standard Deviation (RSD) larger than 30% in QC samples, were discarded. The missing values were replaced with a half of the minimum value found in the data set and the total area normalization for each sample was performed.

The resulting data matrix was then imported into SIMCA-P version 13.0 (Umetrics, Sweden) for subsequent PCA and OPLS-DA analyses. Prior to the multivariate statistical analysis, the data set was log transformed and Pareto scaled. The PCA was used to have an overview of the quality of the data acquisition step, while the OPLS-DA was employed to identify the differential metabolites by calculating the corresponding variable importance in the projection (VIP value). The quality of the model was described by R^2^X, R^2^Y and Q^2^. Excellent models are obtained when the cumulative values of these parameters are greater than or equal to 0.5. Additionally, CV-ANOVA (analysis of variance testing of cross-validated predictive residuals) tests were performed to determine significant differences between groups in the OPLS-DA models. The common practice is to interpret a *p* value lower than 0.05 as denoting a significant model.

### SUS plot

As the control group constituted a common reference for the comparison of the survival and non-survival rats, two OPLS-DA models were computed to distinguish the metabolic patterns of (i) control and survival rats (C vs. S, model 1) and (ii) control and non-survival rats (C vs. NS, model 2). The OPLS-DA models were validated and the statistical parameters for the two models were given in Table 1. One aim of our study was to define metabolites that were specific to non-survival rats. Another aim was to discover the commonly changed metabolites to survival and non-survival rats, which will not be further considered as biomarkers for non-survival rats. SUS plots were constructed with the two OPLS-DA models using the control group as common reference^20^. This plot combines the values of p(corr) (correlation) from the two models in one 2-D plot. As the SUS-plot displays correlation, it should be scaled between −1 and 1 for both axes. To enhance visualization, all metabolites that were found not significant (|p(corr)| <0.5) in either model were removed.

### ASCA/MEBA

The datasets have a multilevel structure which contains multiple types of variation: the dynamic variation of the individuals (the within-individual variation), the static differences between the individuals (the between-individual variation), or combinations thereof. To deal with such a data set, an ANOVA-simultaneous component analysis (ASCA) was applied to split the original dataset into different subsets describing the variation between animals, the variation in time and their interaction^14^. The major advantage of this variation splitting is that each sub model can be analyzed separately without being confounded with the other variation sources. The dataset was first normalized to constant sum and transformed using the generalized log transformation and Pareto scaling, and then introduced into the MetaboAnalyst web portal^23^. Leverage was used to evaluate the importance of the metabolite to the model, and SPE was a test of the fitness of the model for the particular metabolite. Variables with low SPE and higher leverage had a significant contribution to the model and were picked out as influentially affected compounds. Additionally, taking into account any correlations among observations at different times, and the replication, MEBA^16^ was used to identify the metabolites whose expression varied the most across each developmental time point.

### Batch process

Batch process was initially developed for monitoring manufacturing processes, but has proven to be a powerful metabolomics tool in monitoring and interpreting time dependent toxicological responses^17^,^18^. SIMCA-P was used to perform the batch process. The purpose of such process is to diagnose an evolving batch as normal or not, and to obtain indications of variables responsible for any deviations. Each rat is here treated as a separate batch developing with time. The three-way data matrix built up by batches, variables and time was decomposed by subjecting it to two subsequent levels of multivariate modelling.

The idea behind the lower (or observation) level is to unfold the three-way matrix in the batch direction and apply a PLS analysis of the unfolded matrix ((batches × variables) × time) with “time” used as a single y-variable. A model based only on the survival rats was created initially to establish the variability of the biochemical composition of survival samples. Upper and lower control limits corresponding to the ±1.5 standard deviation (SD) of the average for each time point were calculated and used to define the metabolic boundary for “normal” survival samples. Non-survival rats were then predicted into the “survival” model to establish metabolic deviations from the “survival” state. The reason for the deviation can be found in the PLS contribution plots.

The subsequent upper (batch)-level PCA was applied using the lower-level PLS components as input variables, making it possible to view the individual animals as single co-ordinates in multivariate space. Thus at this level clusters and trends in the inter-animal patterns could be detected and interpreted based on data from all included time points. For the interpretation of the detected clustering, it is desirable to use the features of the model hierarchy to trace the interpretation in the upper-level scores and loadings down via the lower level to the original data.

### Multiplex analysis of serum cytokine/chemokine levels

Cytokine/chemokine levels in serum samples obtained at different times following administration of LPS were measured. Concentrations of MIP-1α, IL-1β, IL-6, IL-10, TNF-α and IFNγ were assessed by the Milliplex MAP Rat Cytokine/Chemokine magnetic bead immunoassay kit (RECYTMAG-65K; EMD Millipore, Billerica, MA, USA). All assay plates were run according to the manufacturer’s protocol. Assay results were analyzed using a Luminex 200 (Luminex, Austin, TX, USA) and reported using Luminex xPONENT® software version 3.1.

### Quantification of total thiols in serum

A rapid and easy analytical method was developed for simultaneous detection and quantitation of total thiols in serum based on literatures^44^,^45^. An aliquot of 10 *μ*L serum was spiked with 10 *μ*L internal standard working solution (1 *μ*M diazepam (DZP)). The pH was adjusted to 7 by adding 1 *μ*L of the 1 M ammonium bicarbonate solution, and the mixture was treated with 10 *μ*L of tris(2-carboxyethyl)phosphine hydrochloride (TCEP, 10 mM in 10 mM ammonium bicarbonate solution). After 30 min, 40 *μ*L of N-[P-(2-benzoxazolyl)-phenyl]maleimide solution^45^ (BOPM, 5 mM in acetone) were added, vortex-mixed, and centrifuged at 4 °C for 10 min at 14,000 rpm. Then, the supernatant was transferred and acidified by adding 1 *μ*L of formic acid (25%). 3 *μ*L of the resulting mixture was subjected to LC−MS/MS.

LC−MS/MS was implemented on a Shimadzu Nexera UHPLC system (Shimadzu, Kyoto, Japan) using an Agilent ZORBAX SB-C18 column (100 × 2.1 mm, 1.8 *μ*m (Agilent, MA, USA)) at 40 °C. Elution was performed isocratically with a mobile phase of 70% methanol/water with 0.1% formic acid at a flow rate of 0.2 mL/min over 5 min. The analytes were detected in the positive ion mode using Shimadzu LCMS-8040 triple quadrupole mass spectrometer (Shimadzu, Kyoto, Japan) coupled with an electrospray ionization source. Source and gas parameters for both compounds were: spray voltage 4.5 kV, nebulizer gas 3.0 L/min, drying gas 18.0 L/min, desolvation line temperature 250 °C, heat block temperature 400 °C, CID gas 230 kPa. Collision energy and fragment ions were optimized individually for all analytes (Supplementary Table S8).

## Additional Information

**How to cite this article**: Dai, D. *et al.* Time-resolved metabolomics analysis of individual differences during the early stage of lipopolysaccharide-treated rats. *Sci. Rep.* **6**, 34136; doi: 10.1038/srep34136 (2016).

## Supplementary Material

Supplementary Information

Supplementary Dataset 1

## Figures and Tables

**Table 1:** Results of the cross validation and CV-ANOVA for PCA and OPLS-DA models.

	Model	Component		R^2^X	R^2^Y	Q^2^	*P*-value(CV-ANOVA)
SUS-Plot	2 h C vs. S	OPLS-DA	1 + 1	0.487	0.979	0.953	2.89E-14
	2 h C vs. NS	OPLS-DA	1 + 1	0.443	0.964	0.921	3.14E-18
	6 h C vs. S	OPLS-DA	1 + 1	0.491	0.986	0.945	1.74E-13
	6 h C vs. NS	OPLS-DA	1 + 1	0.472	0.983	0.943	5.06E-20
sub-model of non-survival rats	0 h S vs. NS	PCA	6	0.612		0.0749	
	0 h NS1 vs. NS2	OPLS-DA	1 + 1	0.243	0.913	0.557	3.93E-03
	2 h NS1 vs. NS2	PCA	5	0.604		0.0347	
	2 h NS1 vs. NS2	OPLS-DA	1 + 1	0.525	0.726	0.492	9.19E-03
	6 h NS1 vs. NS2	PCA	2	0.357		0.0672	
	6 h NS1 vs. NS2	OPLS-DA	1 + 1	0.386	0.816	0.449	3.89E-02
