# Supplementary material for: Time-resolved metabolomics analysis of individual differences during the early stage of lipopolysaccharide-treated rats
Source: Sci Rep. 2016 Oct 3;6:34136. doi: 10.1038/srep34136 (PMC5046119; doi:10.1038/srep34136)
Supplement: Supplementary Information [file srep34136-s1.doc]

**Supplementary Materials**

[**Time-resolved metabolomics analysis**](http://www.biochemj.org/content/450/3/595.abstract) **of individual differences during the early stage of lipopolysaccharide-treated rats**

Die Dai^1,2^, Yiqiao Gao^1,2^, Jiaqing Chen^1,2^, Yin Huang^1,2^, Zunjian Zhang^1,2,3*^, Fengguo Xu^1,2,3**^

^1^Key Laboratory of Drug Quality Control and Pharmacovigilance (Ministry of Education), China Pharmaceutical University, Nanjing 210009, China;

^2^Jiangsu Key Laboratory of Drug Screening, China Pharmaceutical University, Nanjing 210009, China

^3^State Key Laboratory of Natural Medicine, China Pharmaceutical University, Nanjing 210009, China

^*^ Corresponding author:

Zunjian Zhang，

Key Laboratory of Drug Quality Control and Pharmacovigilance (Ministry of Education), China Pharmaceutical University, Tongjiaxiang No. 24, Nanjing 210009, China;

Tel/Fax: +86 025 83271454;

E-mail: [zunjianzhangcpu@hotmail.com](mailto:zunjianzhangcpu@hotmail.com)

^**^Corresponding author:

Fengguo Xu，

Key Laboratory of Drug Quality Control and Pharmacovigilance (Ministry of Education), China Pharmaceutical University, Tongjiaxiang No. 24, Nanjing 210009, China;

Tel/Fax: +86 025 83271021;

E-mail: [fengguoxu@gmail.com](mailto:fengguoxu@gmail.com)


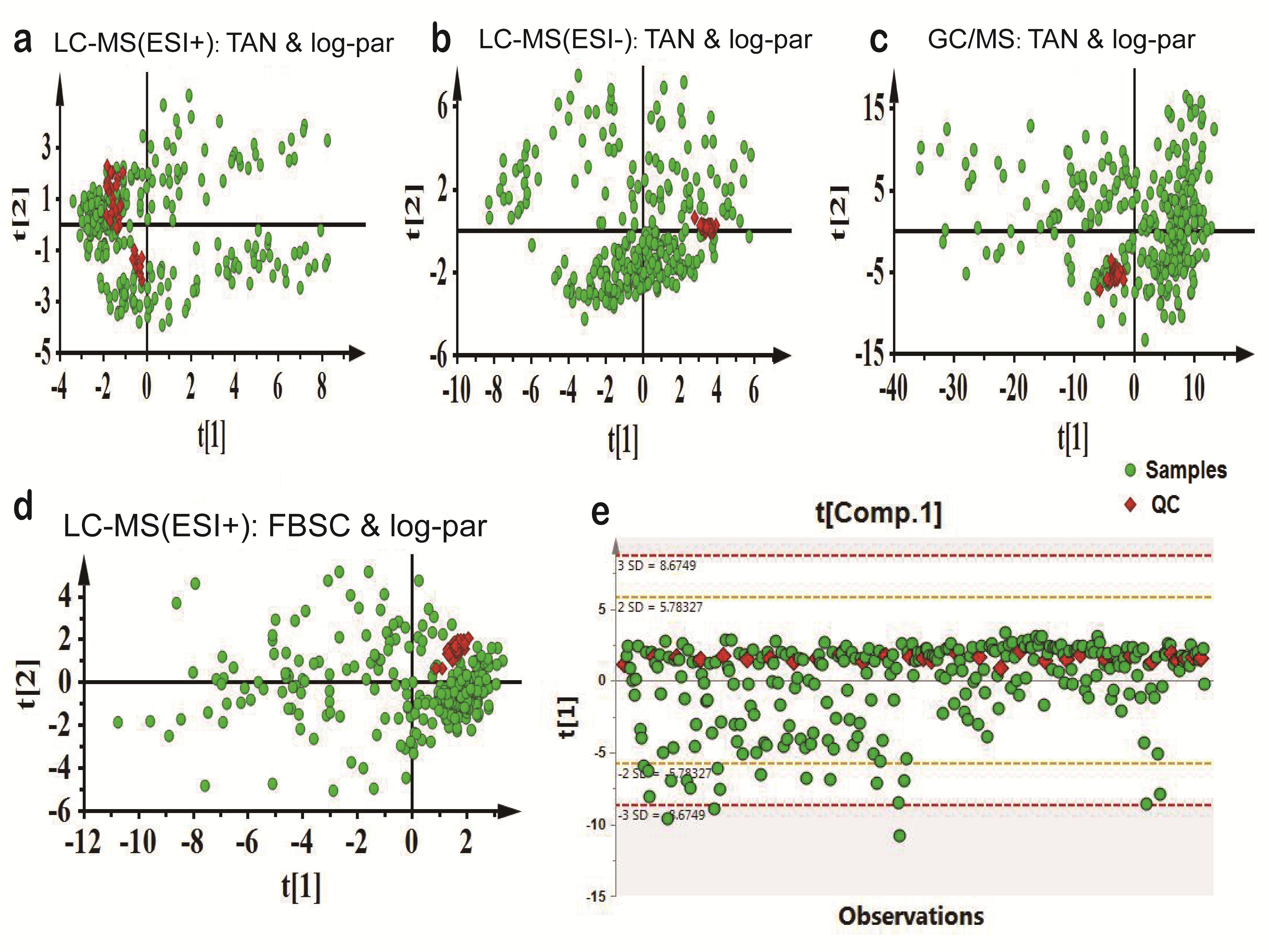


**Figure S1.** PCA scores plots (PC1 vs PC2) of study samples (green) plus QC samples (red) within the run analyzed in LC/MS(ESI+) (**a**), LC/MS(ESI-) (**b**) and GC/MS (**c**) modes after total area normalization (TAN). (**d**) PCA score plot from LC/MS(ESI+) data sets normalized with a feature-based correction algorithm (FBSC), together with time series dependency of the ﬁrst PCA component from the whole analytical run in LC/MS(ESI+) (**e**) mode. Orange and red lines indicate the 2 and 3 SD limits of peak height intensities, respectively. Log-par: the data set was log transformed and Pareto scaled.


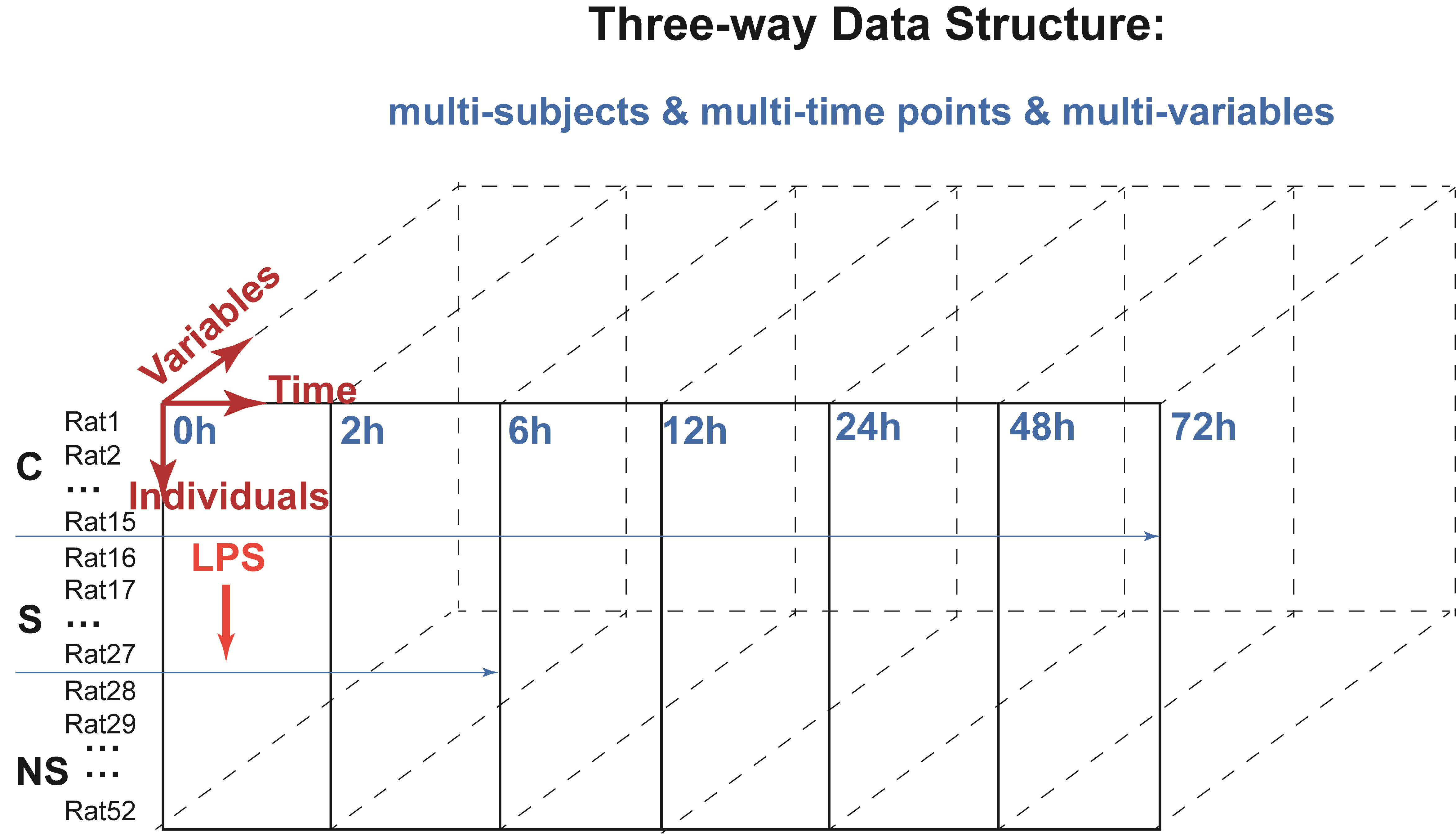


**Figure S2.** The structure of the dataset which is multi-subjects, multi-time points and multi-variables


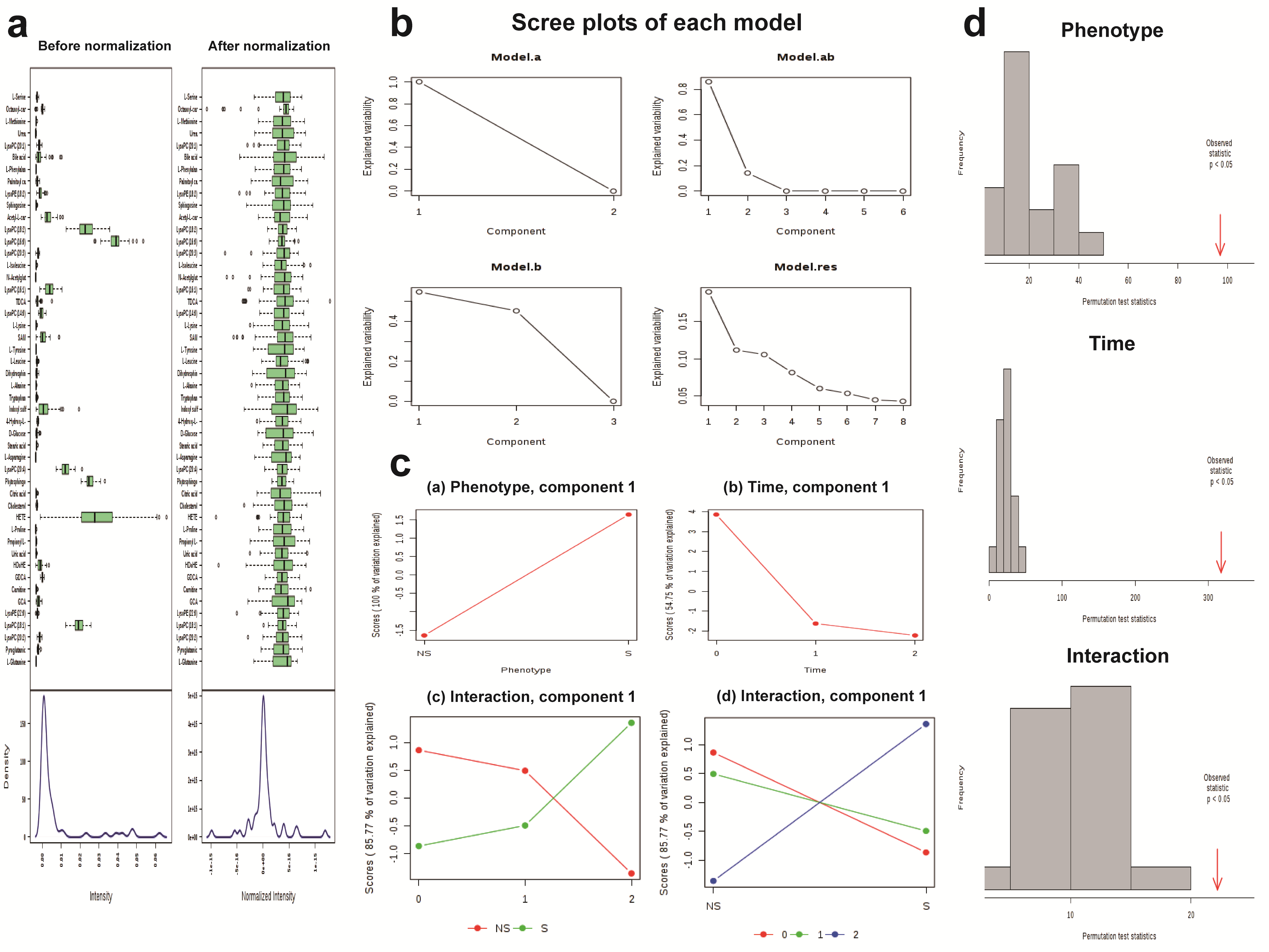


**Figure S3.** (**a**) The effects before and after normalization. (**b**) The scree plots displaying the relationship between eigenvalues and factors. (**c**) ASCA score plots for factor time, factor phenotypes, and their interactions based on PC1 of the corresponding sub models. (**d**) Model validations through permutations, as demonstrated by signiﬁcance levels of *p* < 0.05 for the phenotype, time level, and interaction.


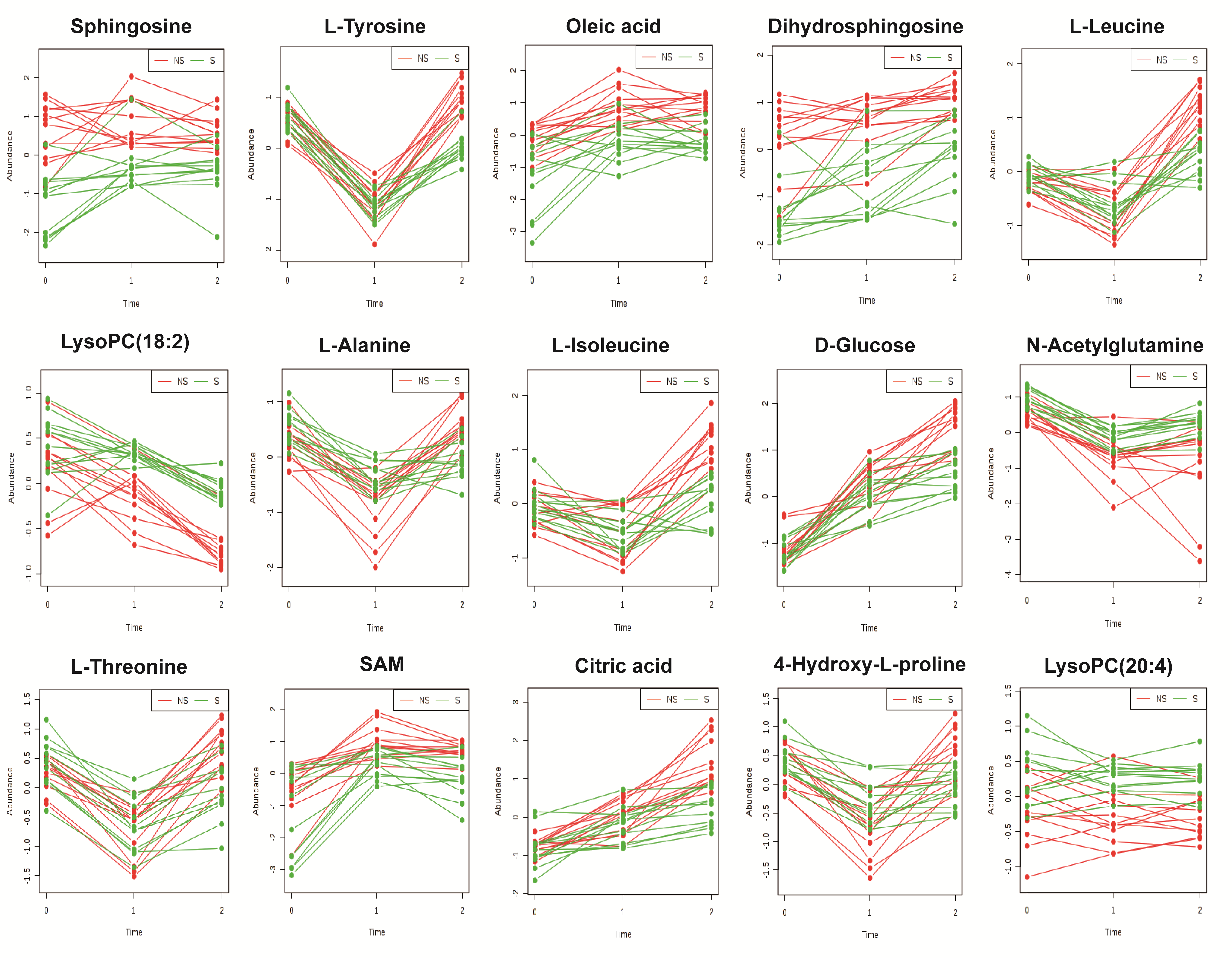


**Figure S4.** Metabolites with distinctive temporal proﬁles identiﬁed by MEBA between survival and non-survival rats within 0h to 6h


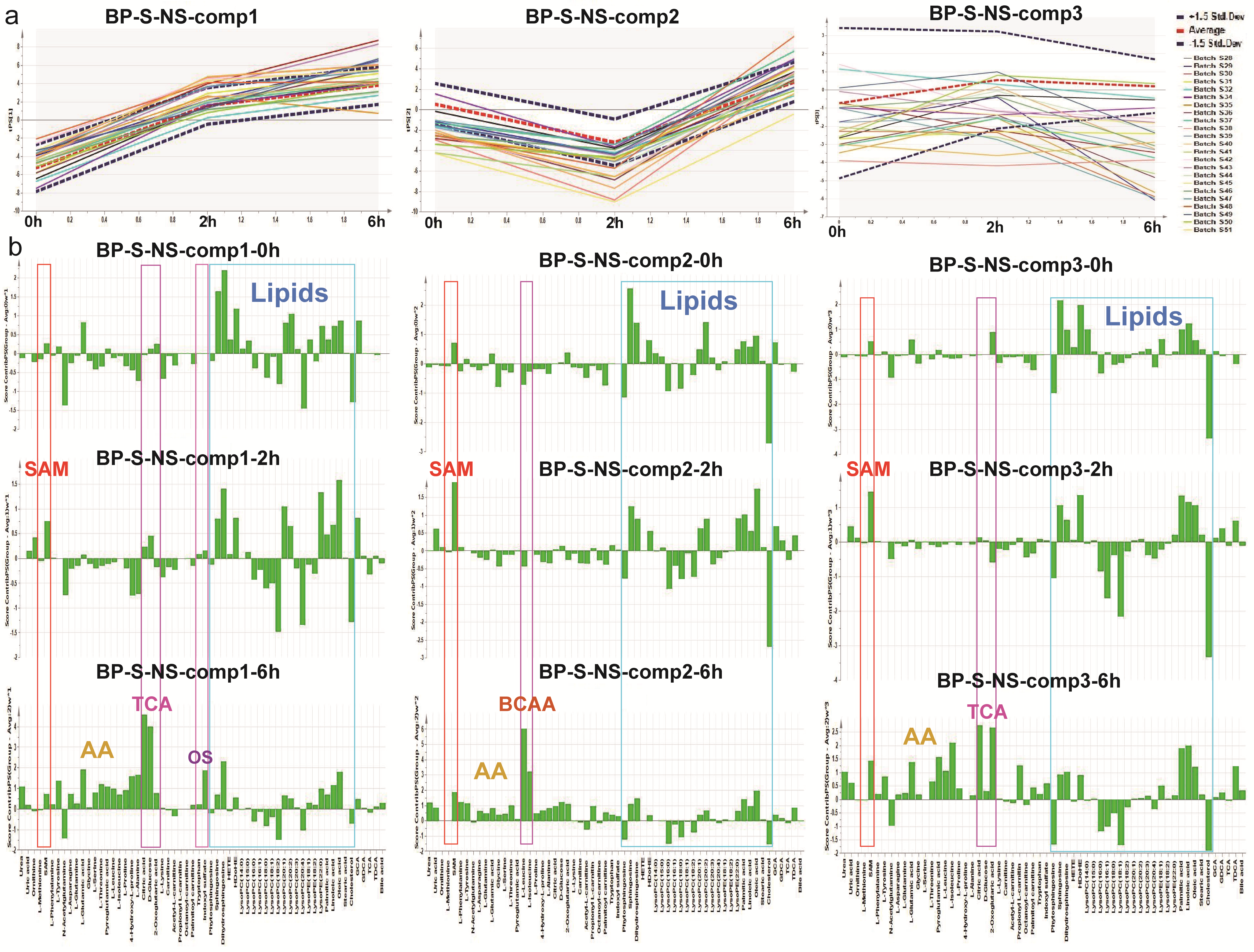


**Figure S5.** (**a**) Lower-level PLS trajectories describing the time course obtained from non-survival rats for PLS components t[1], t[2], t[3]. (**b**) PLS contribution plots for components t[1], t[2], t[3] at 0h, 2h, 6h respectively. AA: amino acids; TCA: metabolites related to TCA cycle; OS: metabolites related to oxidative stress.


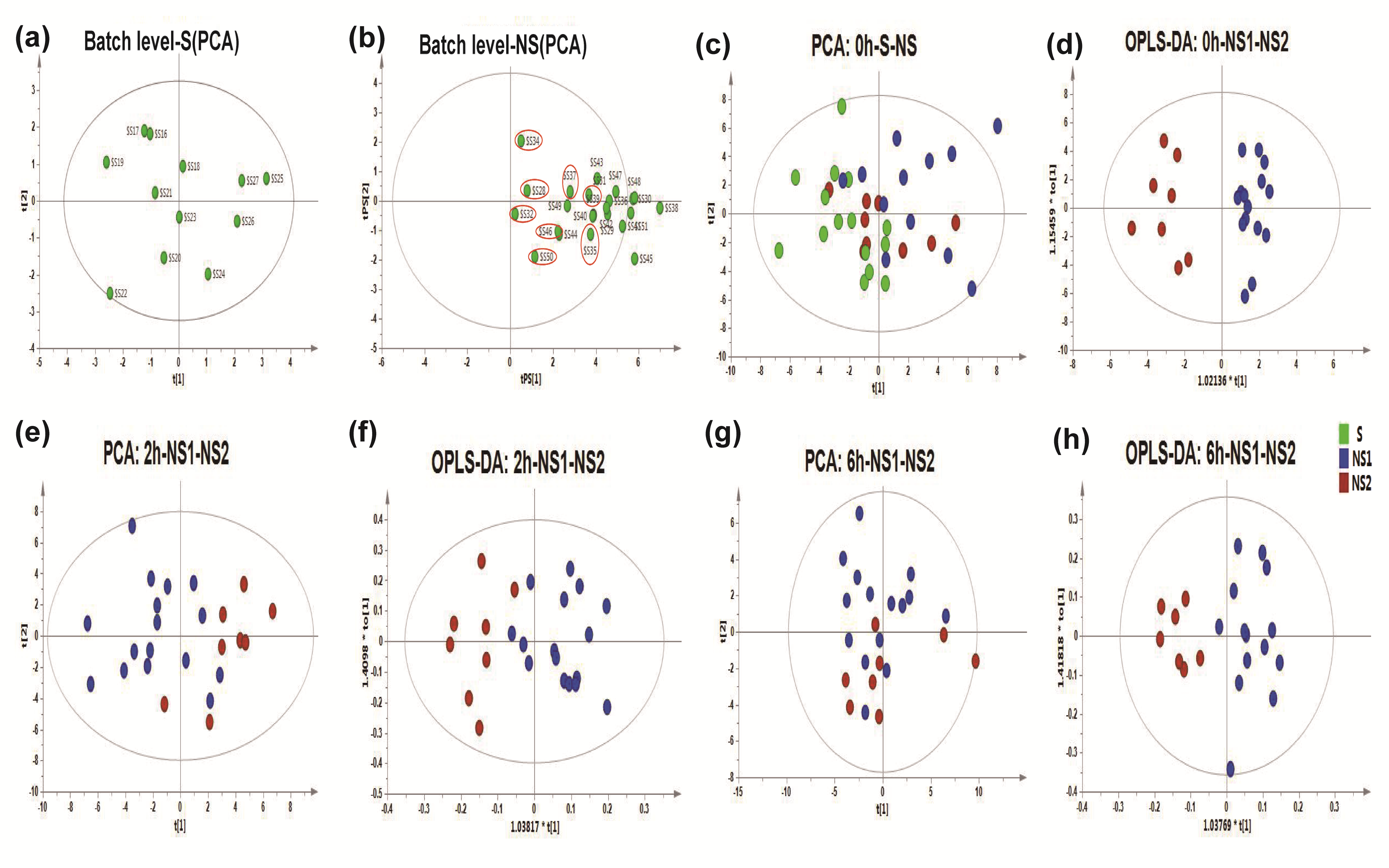


**Figure S6.** Upper-level PCA plot for survival (**a**) and non-survival (**b**) rats, with each rat treated as a single co-ordinate. PCA and OPLS-DA models for the “fast” (NS1group) and the “slow” (NS2 group) responders of the non-survival rats at 0h (**c**, **d**), 2h (**e**, **f**) and 6h (**g**, **h**).


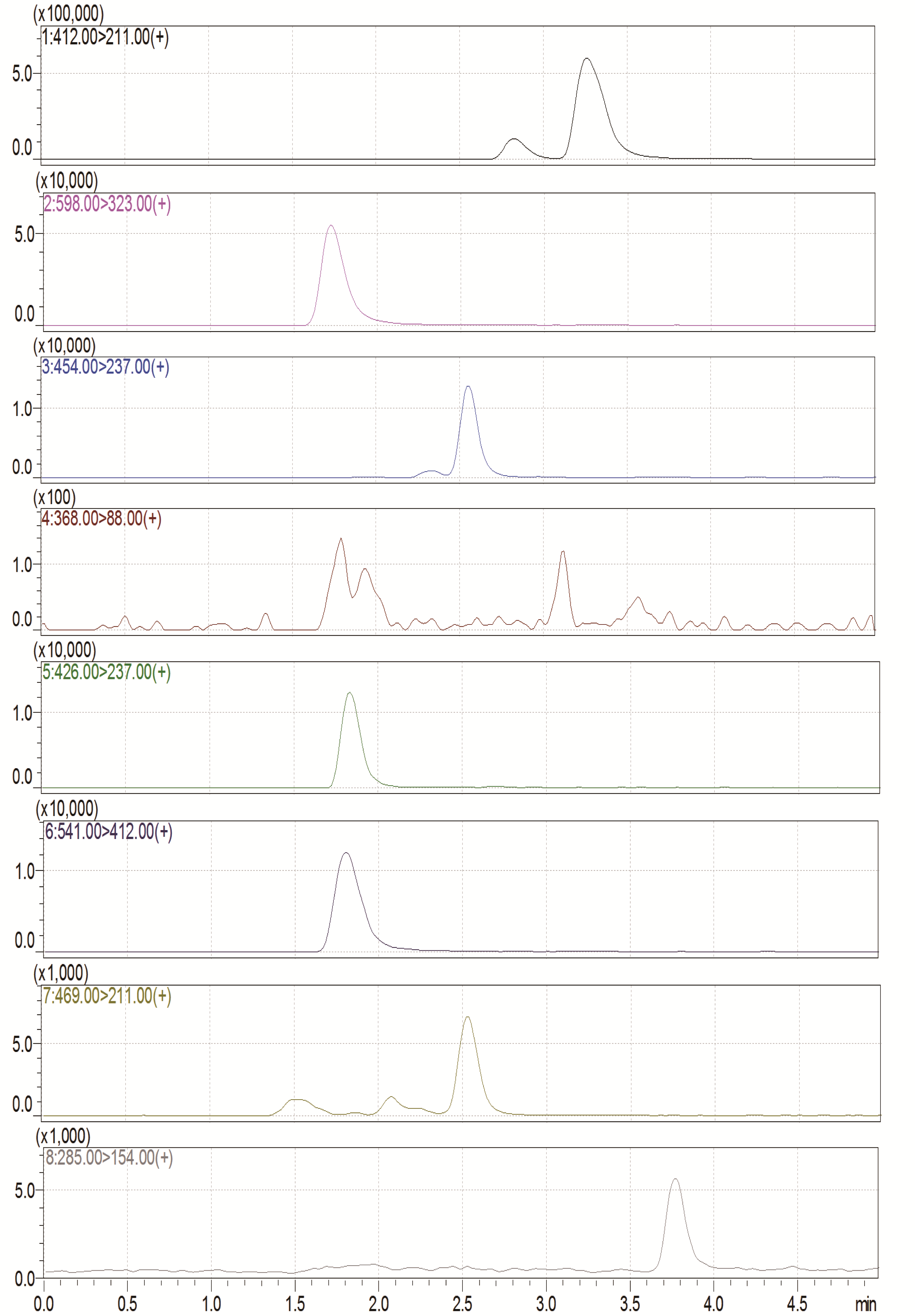


**Figure S7.** Representative MRM chromatogram of each analyte and IS (internal standard) in a serum sample of normal rats.


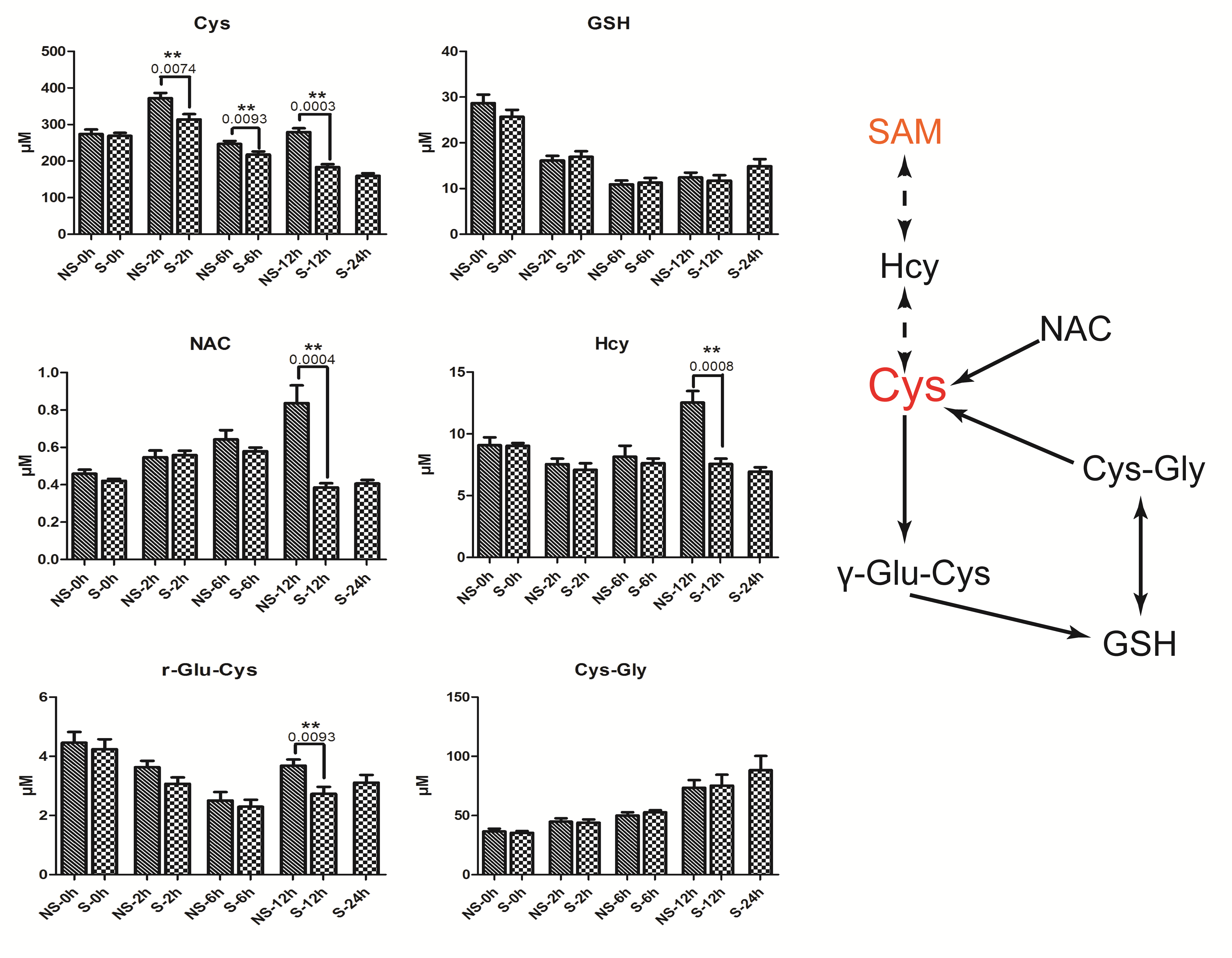


**Figure S8.** Total thiol concentrations determined in survival and non-survival rats during 24h after LPS administration. The nonparametric Mann-Whitney test was employed to assess the statistical significance between S and NS. (n=8) (*): *p*<0.05, (**): *p*<0.01.
